# Biochemical recurrence related metabolic novel signature associates with immunity and ADT treatment responses in prostate cancer

**DOI:** 10.1002/cam4.4856

**Published:** 2022-06-09

**Authors:** Xuan Wang, Zhengtong Lv, Haoran Xia, Xiaoxiao Guo, Jianye Wang, Jianlong Wang, Ming Liu

**Affiliations:** ^1^ Department of Urology Beijing Hospital, National Center of Gerontology, Institute of Geriatric Medicine, Chinese Academy of Medical Sciences Beijing People's Republic of China; ^2^ Graduate School of Peking Union Medical College and Chinese Academy of Medical Sciences Beijing People's Republic of China

**Keywords:** ADT, BCR, metabolism, prognosis, prostate cancer

## Abstract

**Background:**

Prostate cancer (PCa) is a unique cancer from a metabolic perspective. Androgen receptor assumes a vital part in normal and malignant prostate cells regarding almost all aspects of cell metabolism, such as glucose, fat, amino acids, nucleotides, and so on.

**Methods:**

We used The Cancer Genome Atlas database as training set, Memorial Sloan‐Kettering Cancer Center cohort as validation set, and Gene Expression Omnibus database (GSE70769) as test set to identify the optimal prognostic signature. We evaluated the signature in terms of biochemical progression‐free survival (bPFS), ROC curve, clinicopathological features, independent prognostic indicators, tumor microenvironment, and infiltrating immune cells. Nomogram was built dependent on the results of cox regression analyses. GSEA algorithm was used to evaluate differences in metabolism. The signature's prediction of androgen deprivation therapy (ADT) response was validated based on two groups of basic cytological experiments treat with ADT (GSE143408 and GSE120343) and the transcriptional information of pre‐ADT/post‐ADT of six local PCa patients.

**Results:**

We finally input four screened genes into the stepwise regression model to construct metabolism‐related signature. The signature shows good prediction performance in training set, verification set, and test set. A nomogram based on the PSA, Gleason score, T staging, and the signature risk score could predict 1‐, 3‐, and 5‐year bPFS with the high area under curve values. Based on gene‐set enrichment analysis, the characteristics of four genes signature could influence some important metabolic biological processes of PCa and were serendipitously found to be significantly related to androgen response. Subsequently, two cytological experimental data sets and our local patient sequencing data set verified that the signature may be helpful to evaluate the therapeutic response of PCa to ADT.

**Conclusions:**

Our systematic study definite a metabolism‐related gene signature to foresee prognosis of PCa patients which might add to individual prevention and treatment.

## Graphical abstract

Our systematic study established a metabolism‐related gene signature to predict outcomes of PCa patients which may contribute to individual prevention and treatment.

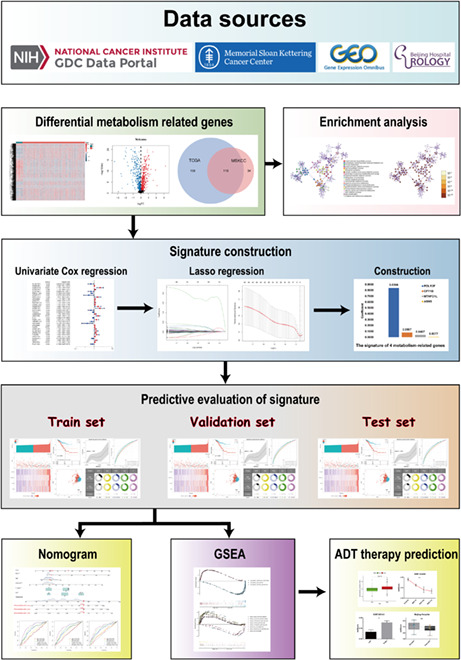

## 1 INTRODUCTION

Prostate cancer (PCa) is still the second most commonly diagnosed malignancy in men all over the world.^1^ As indicated by the latest cancer statistics, it is assessed that roughly 192,000 PCa patients are expected to be diagnosed and in excess of 33,000 passings will happen in the United States in 2020, with a incidence rate of 21% and a mortality rate of 10% in all tumors.^2^ Radical prostatectomy and radiotherapy are the standard treatment for localized PCa, but biochemical recurrences (BCR) still occur in about 20%–40% of patients^3^ and eventually progress to castration‐resistant prostate cancer (CRPC). Without secondary treatment, BCR patients will encounter clinical progression within 5–8 years, and 32%–45% of them will pass on within 15 years.^4^ Active follow‐up and individualized adjuvant therapy, such as androgen deprivation therapy (ADT), chemotherapy, and radiotherapy, should be taken to improve the prognosis and prolong the survival time for these patients. However, traditional clinicopathologic parameters cannot predict prognosis accurately.^5^, ^6^, ^7^ Therefore, the identification of more effective prognostic biomarkers is urgently needed.

Reprogramming of cell metabolism and changes in biological terminology are one of the main characteristics of cancer.^8^ Significant changes in metabolic patterns occur during cell carcinogenesis to meet the abnormal requirements for proliferation and survival, which involve glycolysis, the tricarboxylic acid cycle, oxidative phosphorylation, and metabolism of amino acids, fatty acids, and nucleic acids.^9^ Metabolic parameters, to further develop visualization and draw out endurance. Especially metabolism‐related genes (MRGs), have great value in cancer research. They are not only potential biomarkers for tumor diagnosis, but also play a valuable role in the new mechanisms of control of tumorigenesis.^8^ PCa research also follows this trend, some metabolic pathways responsible for energy production are involved in the pathogenesis and progression of PCa by disabling the regulation of cell proliferation and growth, triggering resistance to treatment.^10^ It presents unique basic metabolic changes in the process of development and progression, like glucose, lipids, and glutamine to maintain its cell survival and growth.^11^ Therefore, it is vital to clarify the relationship between metabolism‐related genes and PCa. However, the value of metabolic markers in the prognosis of PCa remains unclear.

**FIGURE 1:**
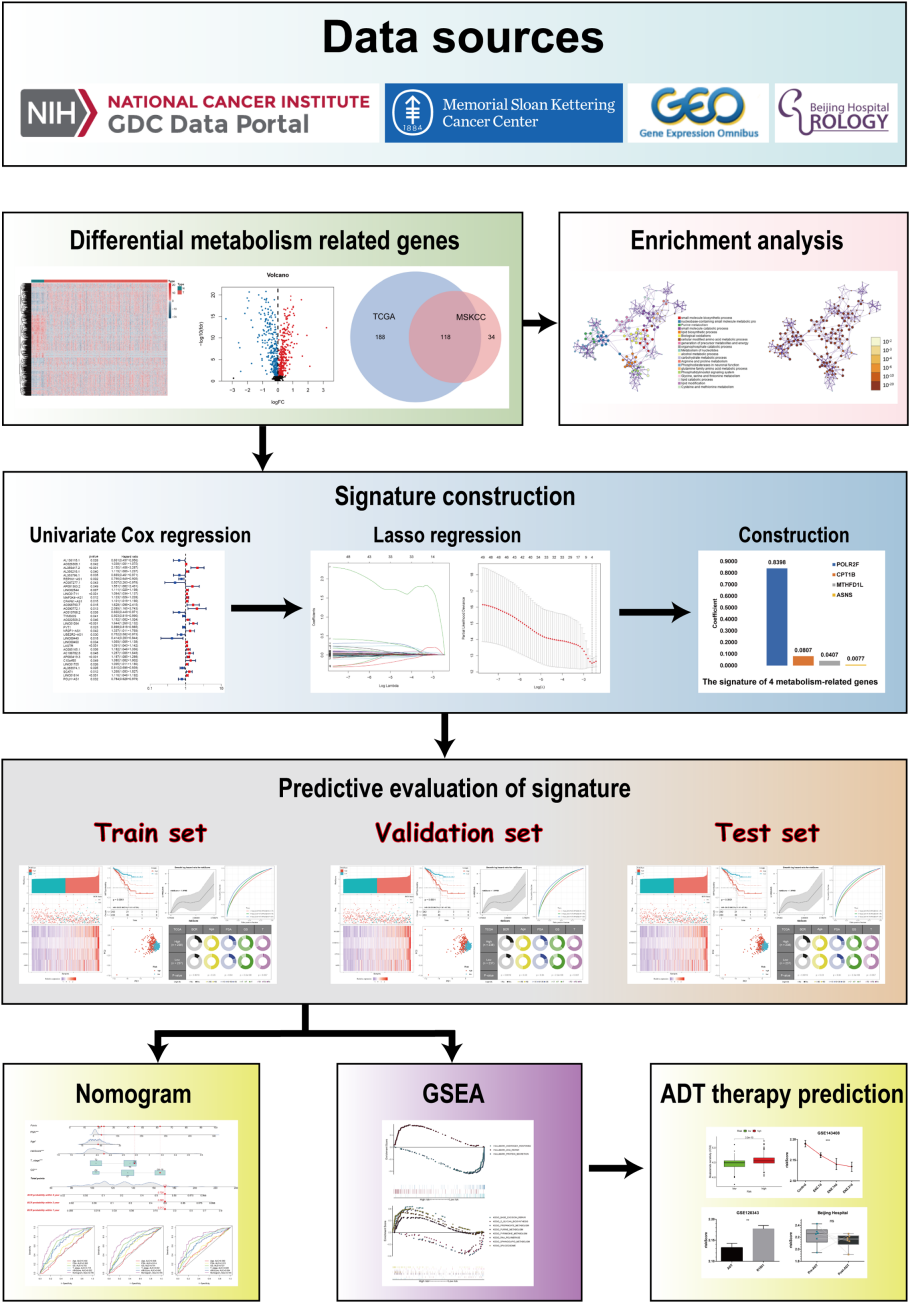
Study flow chart.

With the expanding use of bioinformatics in the diagnosis and prognosis of malignant tumors, a few scientists have connected metabolomics and genomics to explore the relationship with tumors. In this study, we integrated transcriptome and clinicopathological information from The Cancer Genome Atlas (TCGA), Memorial Sloan‐Kettering Cancer Center (MSKCC), and Gene Expression Omnibus databases (GSE70769). TCGA cohort was used as training set to construct four MRGs signature, MSKCC cohort as training set, and GSE70769 cohort as test set, which showed good prognosis prediction performance. In addition, the underlying mechanism behind the signature is verified by the basic experimental data set and our local hospital patient sequencing data set. The general idea of the study is shown in Figure 1. We believe that our results can give new bits of knowledge into the metabolic mechanism of PCa and help to explore the prognostic worth of this signature in patients with PCa.

## 2 MATERIALS AND METHODS

### 2.1 Data Collection

All data information was showed in Table S1. We downloaded transcriptome profiling data in fragments per kilobase million (FPKM) format of 489 PCa patients and 51 normal tissues, along with the corresponding clinical information from the TCGA database (https://portal.gdc.cancer.gov/), normalized log2 mRNA expression data of 150 PCa patients and 29 normal tissues, along with the corresponding clinical information from the MSKCC database (http://cbio.mskcc.org/cancergenomics/prostate/), and expression profiling of 94 PCa patients along with the corresponding clinical information from the GEO database (GSE70769)^12^ (https://www.ncbi.nlm.nih.gov/geo/). The batch effect was removed with R package “sva.” Two sequencing datasets (GSE143408^13^ and GSE120343^14^) involving basic experiments in PCa LNCaP cells were also downloaded for mechanism verification. Finally, six paired pre‐ and post‐ADT PCa lesions from our local hospital were obtained from patients receiving neoadjuvant ADT for RNA‐seq analysis. Sequencing data has been uploaded to the GEO platform (GSE150368).^15^ It was approved by the ethics committee at Beijing Hospital (2018BJYYEC‐085‐03) and informed consent was obtained from all patients.

### 2.2 Differential MRGs acquisition and functional enrichment analysis

Firstly, 937 MRGs were obtained based on KEGG pathway‐related gene set “c2.cp.kegg. v7.4. symbols.“ The significant differential expression of MRGs (DEGs) were identified by R package “limma” with log2 FC >1.0 and a FDR of 0.05. To investigate the potential functional implication of these MRGs, all DEGs were further analyzed by Metascape.^16^

### 2.3 Establishment of the prognosticate signature

The intersection of TCGA and MSKCC differential genes was included in subsequent univariate Cox regression analysis to obtain MRGs related to biochemical progression‐free survival (bPFS). Subsequently, the least absolute shrinkage and selection operator (LASSO) and Cox regression analysis were performed to single out the ideal prognostic MRGs. Finally, considering the optimization of gene expression, the risk scoring formula is obtained: Risk‐score = (exp MRG1 × coef MRG1) + (exp MRG2 × coef MRG2) + … + (exp MRGn × coef MRGn).

### 2.4 Evaluation of constructed signature

Taking the median risk score of the training set as the threshold, the eligible PCa patients were partitioned into low‐ and high‐risk groups. Kaplan Meier curves were plotted to estimate the bPFS. Smooth HR Curves showed the nonlinear relationship between risk score and bPFS. The R package “survival ROC” was used to conduct ROC analysis. Principal component analysis (PCA) showed the visualization of signature gene for distinguishing two groups of patients. Univariate and multivariate Cox regression analyses were utilized to determine independent prognostic significances of the signature.

### 2.5 Construction of the nomogram

The “rms” and “regplot” R packages were used to construct the nomogram, and the predictors in the multivariate COX analysis that were significantly related to bPFS were fitted into the nomogram, to predict bPFS in PCa patients more accurately. Moreover, the reliability of the nomogram was assessed utilizing the concordance index (C‐index) and ROC curves of 1‐, 3‐, and 5‐year bPFS rate were to determine the prognostic value of the nomogram.

### 2.6 Immune characteristics analysis

The immune, stromal, and estimate scores were assessed by using the “estimate” R package. The scores were utilized to mirror the degree of immune cell and stromal cell infiltration of tumor tissue. To explore the correlation between the risk score and immune cell infiltration, we used the R software package “CIBERSORT” to draw the immune cell infiltration matrix. The anti‐cancer immunity of the seven‐step cancer immune cycle was as follows: Step 1 ‐ release of cancer cell antigens, Step 2 ‐ cancer antigen presentation, Step 3 ‐ priming and activation, Step 4 ‐ trafficking of immune cells to tumors, Step 5 ‐ infiltration of immune cells into tumors, Step 6 ‐ recognition of cancer cells by T cells and Step 7 ‐ killing of cancer cells. We downloaded these seven‐step cancer immunity cycle information for each sample of PCa in TCGA (http://biocc.hrbmu.edu.cn/TIP/).

### 2.7 Gene set enrichment analysis between different risk groups

GSEA programming was utilized to find the differences between the high‐ and low‐risk groups in the Hallmark and KEGG pathway (https://www.GSEA‐msigdb.org/). Nominal *p* value <5% was considered significant.

### 2.8 Statistical analysis

All statistical analyses were performed utilizing the Software R version 4.1.0. The Fisher's exact test or Chi‐squared test was utilized for categorical variables, and the Wilcoxon rank‐sum test or Student *t*‐test was utilized for continuous variables. Pearson correlation test was utilized to evaluate the correlation between the two groups of data. Two‐sided *p* value <5% were considered significant for the whole statistical analyses.

## 3 RESULT

### 3.1 Identification of differential MRGs expression profiles

**FIGURE 2:**
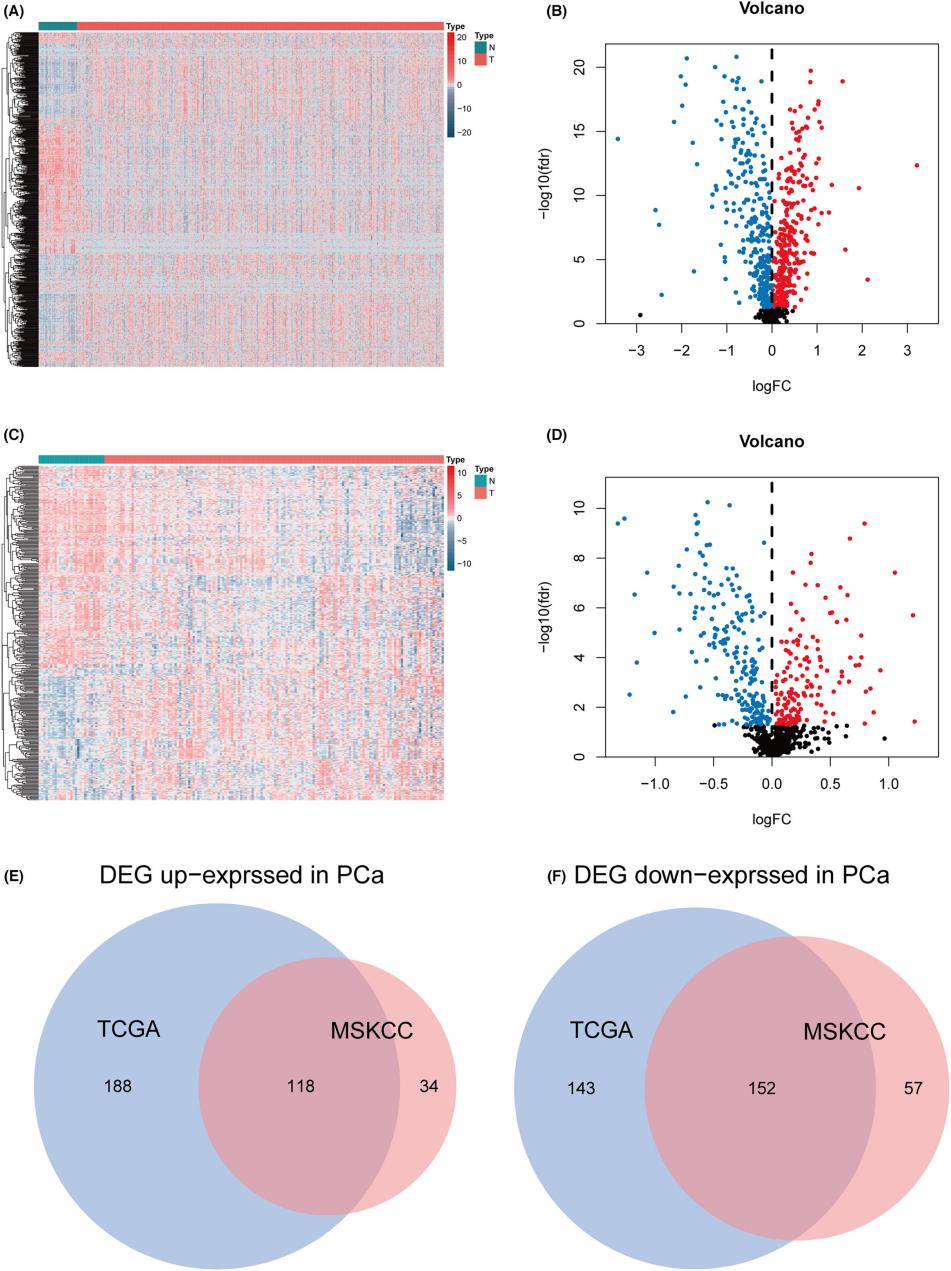
Screening for metabolism‐related genes. (A) Heatmap display of MRGs in TCGA cohort. (B) Volcano map shows the different MRGs in TCGA cohort. (C) Heatmap display of MRGs in MSKCC cohort. (D) Volcano map shows the different MRGs in MSKCC cohort. (E) Venn diagram of up‐regulated MRGs in two cohorts. (F) Venn map of down‐regulated MRGs in two cohorts.

Through difference analysis, among 601 DEGs in TCGA data set, 306 genes were up‐regulated, and 295 genes were down‐regulated (Figure 2A,B) (Table S2). In MSKCC dataset, 361 metabolism‐related DEGs were dysregulated, including 152 up‐regulated and 209 down‐regulated DEGs (Figure 2C,D) (Table S3). We intersected these genes in the TCGA dataset and the MSKCC dataset, and finally obtained a total of 118 DEGs that were jointly up‐regulated and 152 DEGs that were jointly down‐regulated (Figure 2E,F).

### 3.2 Functional enrichment of these 270 DEGs

**FIGURE 3:**
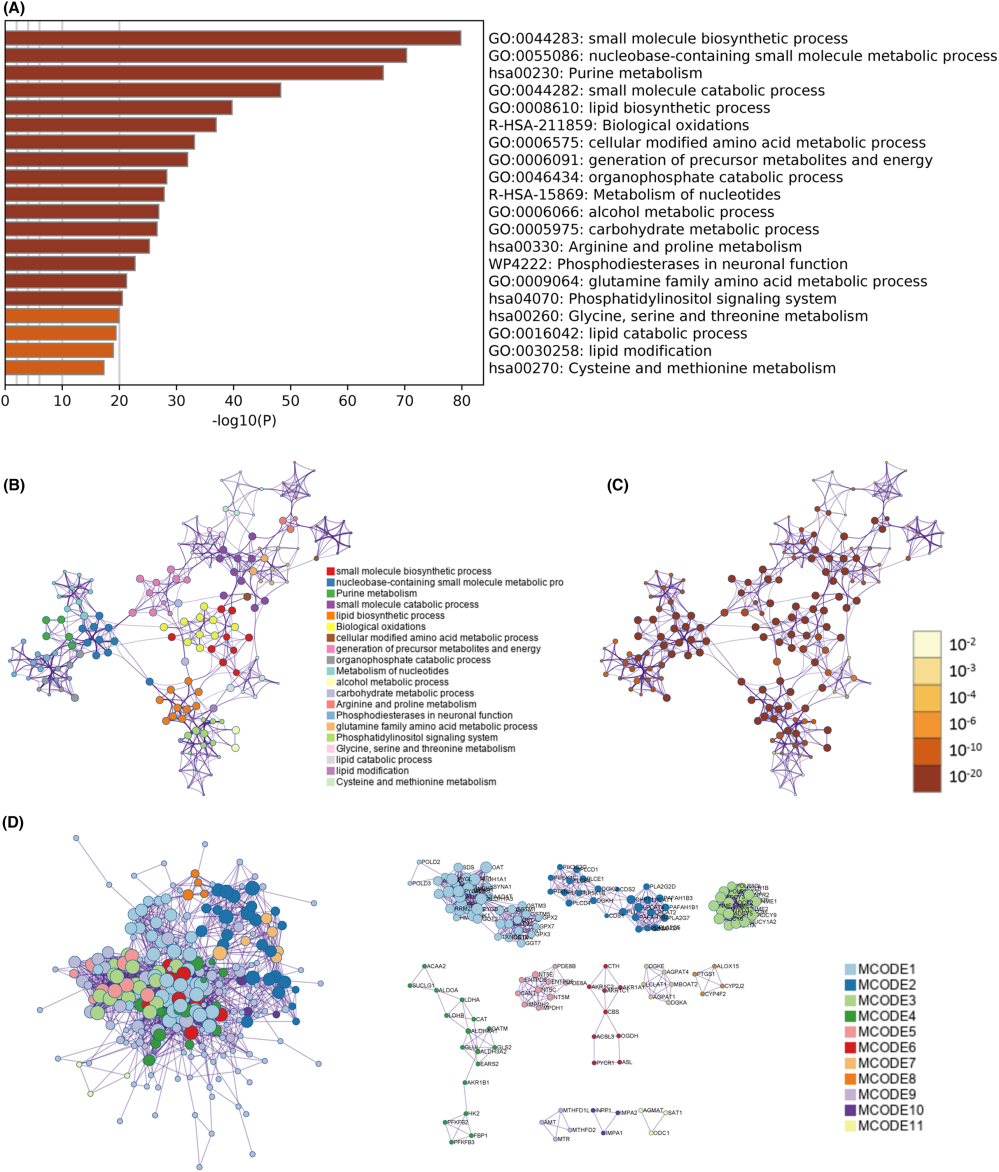
Functional enrichment of these 270 DEGs. (A) Bar graph of enriched terms across 270 DEGs, colored by p‐values. (B) Network of enriched terms colored by cluster ID, where nodes that share the same cluster ID are typically close to each other. (C) Network of enriched terms colored by p‐value, where terms containing more genes tend to have a more significant *p*‐value. (D) Protein–protein interaction network and MCODE components identified.

The functional enrichment results by Metascape showed that these genes were principally concerned in metabolism‐related pathway, such as small molecule biosynthetic process, nucleobase‐containing small molecule metabolic process, purine metabolism, small molecule catabolic process, lipid biosynthetic process, etc (Figure 3A–C) (Table S4). For these 270 DEGs, protein–protein interaction enrichment analysis has been completed with the following databases: STRING, BioGrid, OmniPath, InWeb_IM. The Molecular Complex Detection (MCODE) algorithm has been applied to distinguish densely connected network components.^17^ 11 MCODE components were identified, including glutathione, glycerophospholipid, purine, fructose, and mannose metabolism, etc (Figure 3D) (Table S5). These findings suggest that the differential expression of these DEGs in individuals may lead to different metabolic phenotypes.

### 3.3 Construction of a prognostic MRG signature

**FIGURE 4:**
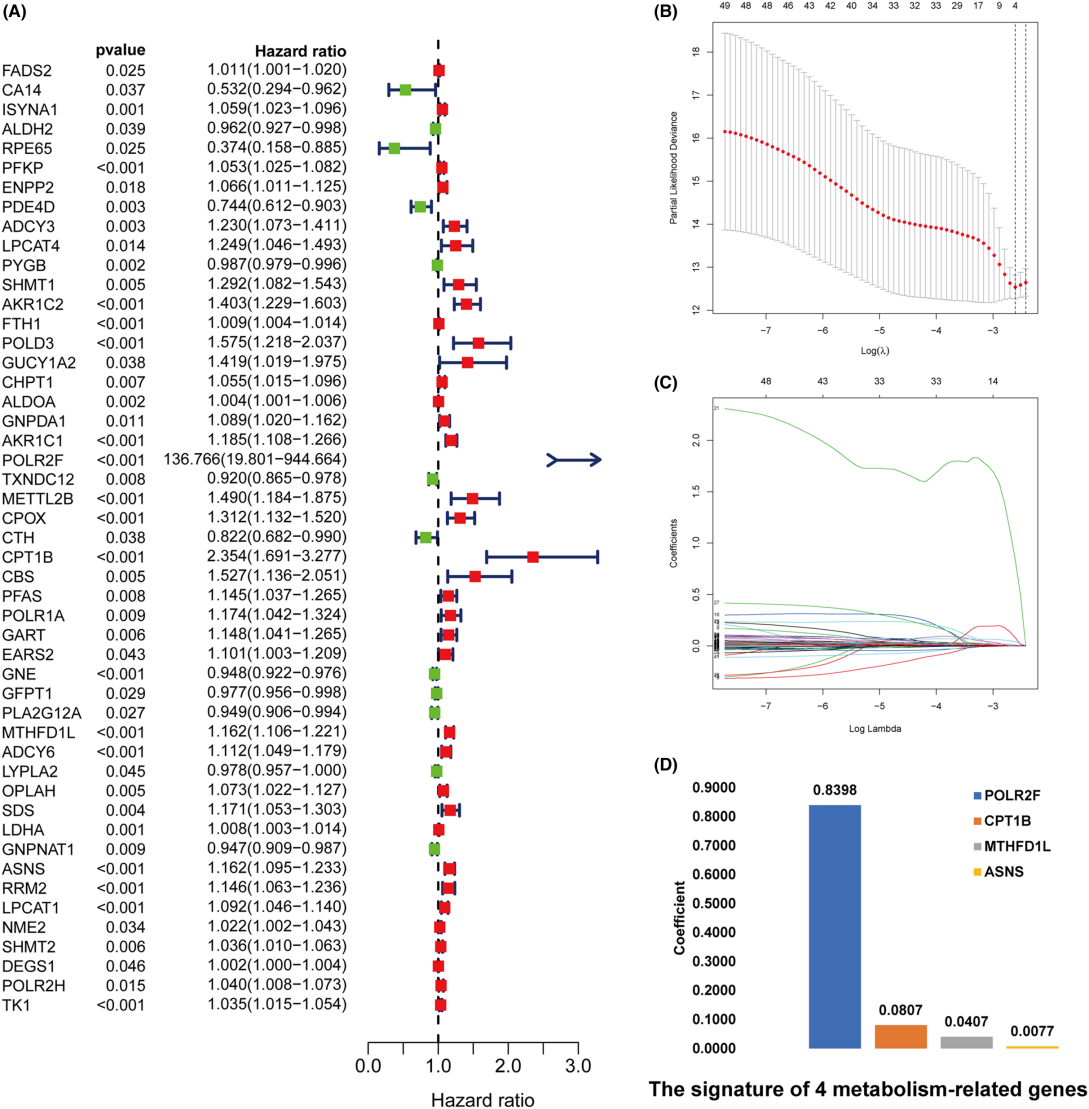
Construction of a prognostic MRG signature. (A) univariate Cox regression analysis to determine that 49 DEGs were associated with BCR of PCa. (B) The coef reach zero in some genes when the lambda value increases, and this indicates that those genes have no effect on the model. (C) The deviance of tenfold cross‐validation obtained four prognostic genes. The best model depends on the minimum value of partial likelihood deviance. (D) Four MRGs prognostic hub genes with corresponding coefficients.

First, we used univariate Cox regression analysis to determine 49 DEGs were related with BCR of PCa (Figure 4A) (Table S6). Next, to avoid over fitting, a LASSO and Cox regression model was utilized to work out the most valuable prognostic genes, bringing about a signature with four genes: POLR2F, CPT1B, MTHFD1L, and ASNS (Figure 4B–D). The stepwise Cox proportional risk regression model showed that the 4‐gene signature was the ideal model. It is worth noting that all these four genes were risk factors.

### 3.4 Predictive value of the signature in train set and validation set

**FIGURE 5:**
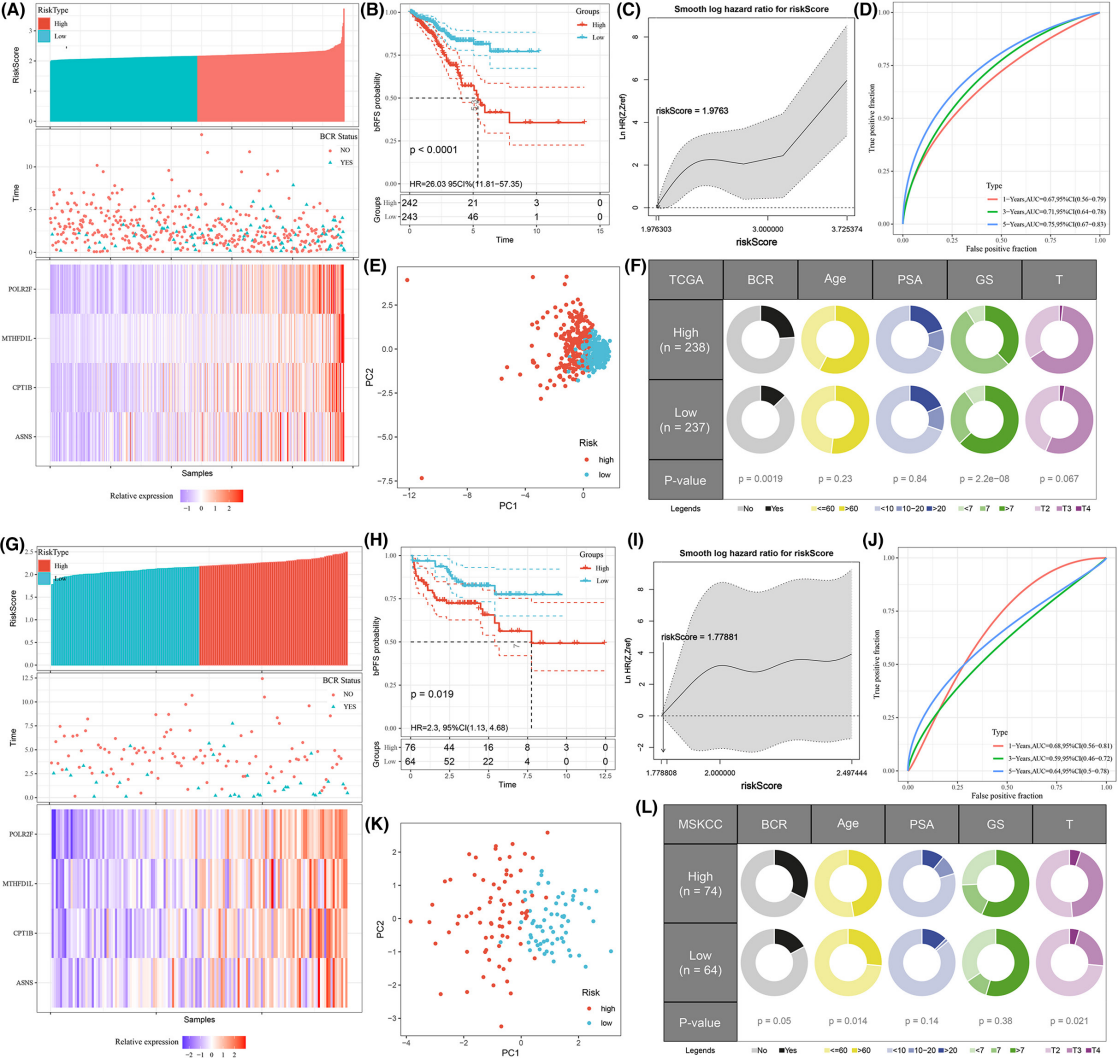
Predictive value of the signature in train set and validation set. (A) The distribution of risk scores (upper panel), the distribution of BCR time (middle panel), and four‐gene expression profiles (bottom panel) in TCGA cohort. (B) Kaplan–Meier survival analysis of the signature in TCGA cohort. (C) Nonliner‐dependent effect of risk score on bPFS in TCGA cohort. (D) Time‐dependent ROC analysis of the risk signature in TCGA cohort. (E) PCA analysis shows a two‐dimensional distinction between high‐ and low‐risk groups in TCGA cohort. (F) Relationship between risk score and clinicopathologic features in TCGA cohort. (G–L) The same statistical method was used to evaluate the performance of signatures in the validation set (MSKCC).

Each patient was then scored based on this signature. The median risk score separated all PCa patients into low‐ and high‐ risk groups in train set (TCGA). The dispersion of risk scores, the BCR status, and the heatmap of the 4 prognostic genes are shown in Figure 5A. Kaplan–Meier curves displayed that PCa patients with the high‐risk had a significantly poorer bRFS compared to those in the low‐risk one (*p* < 0.0001) in the training database (Figure 5B). Taking into account the non‐linear impact of the risk score on BCR risk, the smoothHR curve shows that as the cutoff value of the selected risk score increases, so does the HR (Figure 5C). Areas under the curve value of the signature predicting the 1‐, 3‐ and 5‐year bPFS rates were 0.67, 0.71, and 0.75, indicating that this prognostic model exhibited a good sensitivity and specificity (Figure 5D). PCA showed that two‐dimensional spatial distribution of patients in different risk groups was different (*p* < 0.001) (Figure 5E). The clinicopathological information of the patients are described in Figure 5F, and high‐risk score was related to higher BCR probability, higher Gleason score (GS), and worse T staging.

According to the same cut‐off value determined in train set, the same analyses were further performed in an external validation set (MSKCC). The 140 patients were divided into 76 high‐risk and 64 low‐risk groups. Figure 5G showed the risk score distribution, BCR status, and Heatmap of the four prognostic genes. Kaplan–Meier curves also displayed that PCa patients in the high‐risk group had a significantly poorer bRFS compared to those in the low‐risk one (*p* = 0.019) in the validation database (Figure 5H). With the increase of cutoff value of the selected risk score, HR also increased (Figure 5I). Areas under the curve value of the signature predicting the 1‐, 3‐ and 5‐year bPFS rates were 0.68, 0.59, and 0.64 (Figure 5J). PCA showed that two‐dimensional spatial distribution of patients in different risk groups was also different (*p* < 0.001) (Figure 5K). The clinicopathological information of the patients are described in Figure 5L, and high‐risk score was related to higher BCR probability, older age, and worse T staging.

### 3.5 Identification of independent prognostic parameter and nomogram construction

**FIGURE 6:**
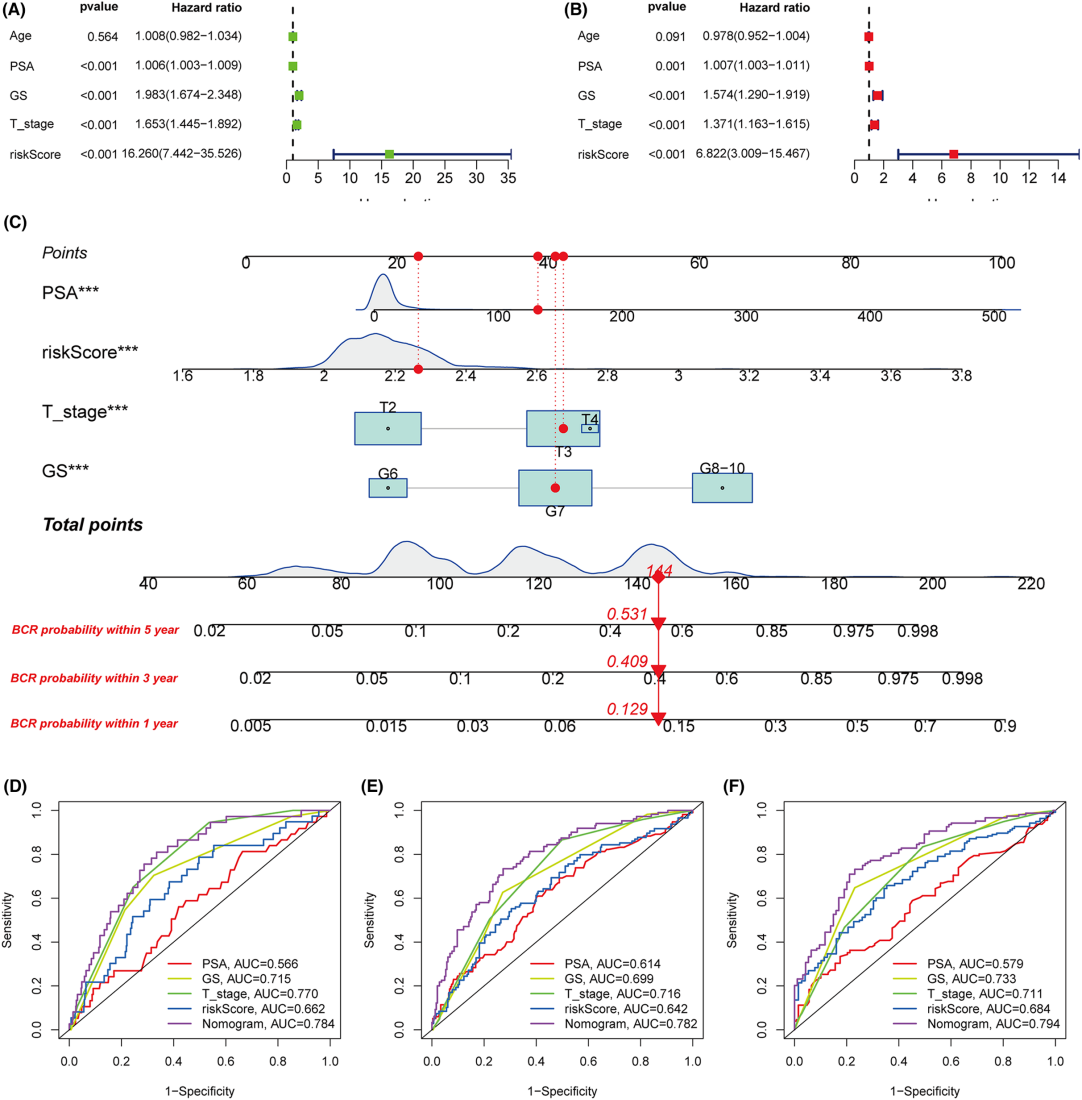
Identification of independent prognostic parameter and nomogram construction. (A) Univariate Cox regression analysis of bPFS. (B) Multivariate Cox regression analysis of bPFS. (C) A prognostic nomogram including signature risk score and other clinical factors. For example, a patient with an initial PSA 132 ng/ml, a GS score of 7, a T stage of T3, and a signature score of 2.265 had a total nomogram score of 144, implying the 1‐year, 3‐year, and 5‐year BCR probability of 12.9%, 40.9%, and 53.1%, respectively. (D–F) ROC curve used to evaluate the 1‐, 3‐, and 5‐year bPFS predictive efficiency.

When we combine the patients in the train set (TCGA) and the validation set (MSKCC), Univariate and multivariate Cox regression analyses were performed to assess the prognostic significance of this signature in combination with various clinical parameters (625 PCa patients). Through univariate and multivariate Cox regression analyses, we found four independent prognostic factors, including PSA (HR = 1.007, 95% CI = 1.003–1.011, *p* = 0.001), GS (HR = 1.574, 95% CI = 1.290–1.919, *p* < 0.001), T staging (HR = 1.371, 95% CI = 1.163–1.615, *p* < 0.001), and risk score (HR = 6.822, 95% CI = 3.009–15.467, *p* < 0.001) (Figure 6A,B). Since regression analysis confirmed that PSA, GS, T staging, and risk score were independent risk factors for PCa, we constructed a nomogram based on these variables to estimate the probability of 1‐, 3‐, and 5‐year bPFS (Figure 6C). the C‐index of the nomogram was 0.759, and the AUC values predicting 1‐year, 3‐year, and 5‐year BCR were 0.784, 0.782, and 0.794, respectively, which was higher than any other clinical parameter (Figure 6D–F).

### 3.6 Performance of the signature and nomogram in test set

**FIGURE 7:**
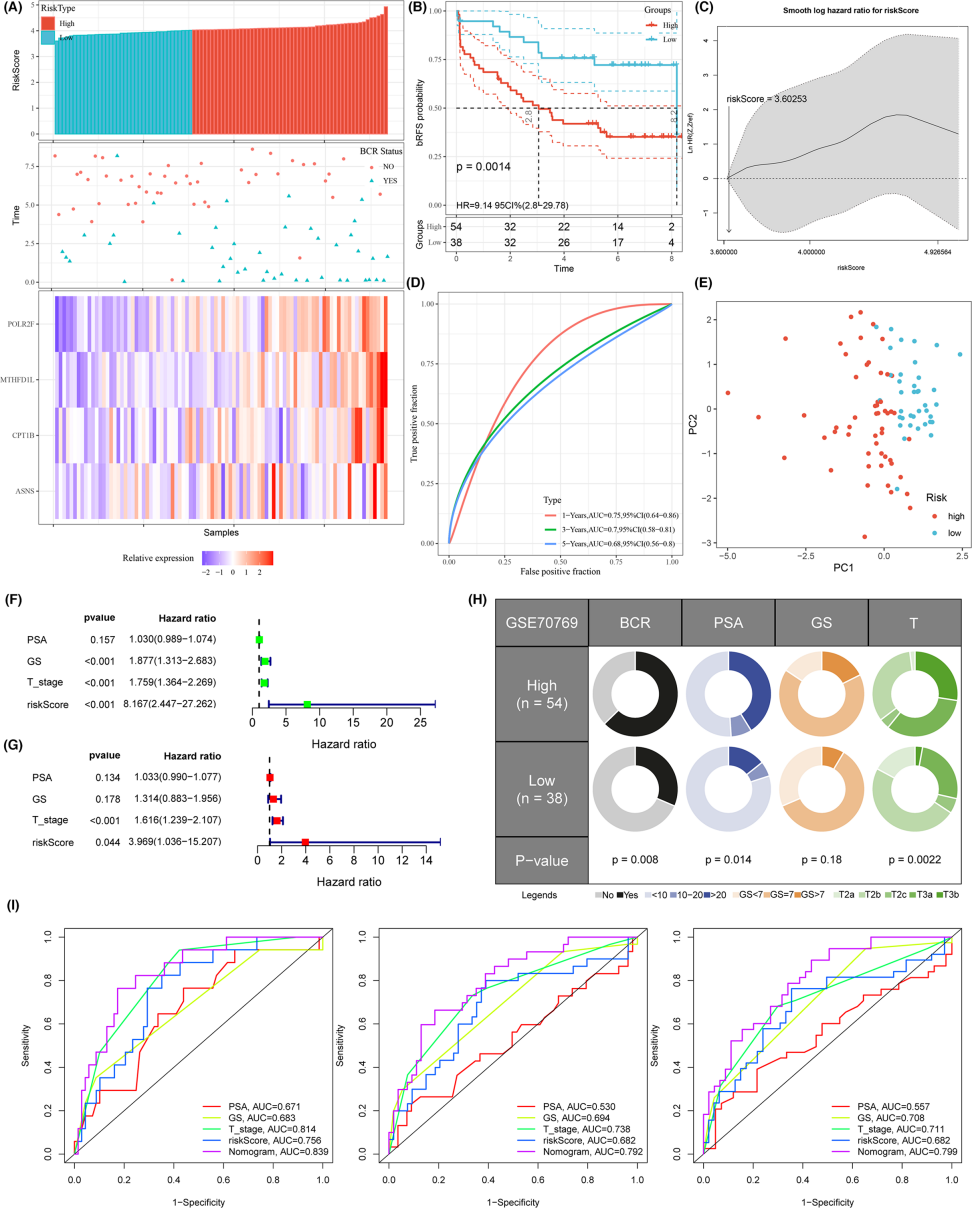
Performance of the signature and nomogram in test set (GSE70769). (A) The distribution of risk scores (upper panel), the distribution of BCR time (middle panel), and four‐gene expression profiles (bottom panel). (B) Kaplan–Meier survival analysis of the signature. (C) Nonliner‐dependent effect of risk score on bPF. (D) Time‐dependent ROC analysis of the risk signature. (E) PCA analysis shows a two‐dimensional distinction between high‐ and low‐risk groups. (F) Univariate Cox regression analysis of bPFS. (G) Multivariate Cox regression analysis of bPFS. (H) Relationship between risk score and clinicopathologic features. (I) ROC curve used to verify the 1‐, 3‐, and 5‐year bPFS predictive efficiency of nomogram.

A good prognosis model should be robust in multiple independent data sets. A group of 92 patients with complete clinical information served as an external test set (GSE70769),^12^ which was evaluated using the same set of methods. The 92 patients were divided into 54 high‐risk and 38 low‐risk groups. Figure 7A showed the risk score distribution, BCR status, and Heatmap of the four prognostic genes. Kaplan–Meier curves also displayed that PCa patients in the high‐risk group had a poorer bRFS compared to those in the low‐risk one (*p* = 0.0014) (Figure 7B). With the increase of cutoff value of the selected risk score, HR also increased (Figure 7C). Areas under the curve value of the signature predicting the 1‐, 3‐ and 5‐year bPFS rates were 0.75, 0.70, and 0.68 (Figure 7D). PCA also showed that two‐dimensional spatial distribution of patients in different risk groups was also different (*p* < 0.001) (Figure 7E). The signature remained an independent prognostic indicator for PCa patients in test set through the univariate and multivariate analysis (Figure 7F,G). The high‐risk score was related to higher BCR probability, higher PSA, and worse T staging (Figure 7H). The nomogram constructed above was applied to this test data set and also showed good prediction performance. The AUC values predicting 1‐year, 3‐year, and 5‐year BCR were 0.839, 0.792, and 0.799, respectively (Figure 7I).

### 3.7 Immune characteristics of patients in different risk score patient

**FIGURE 8:**
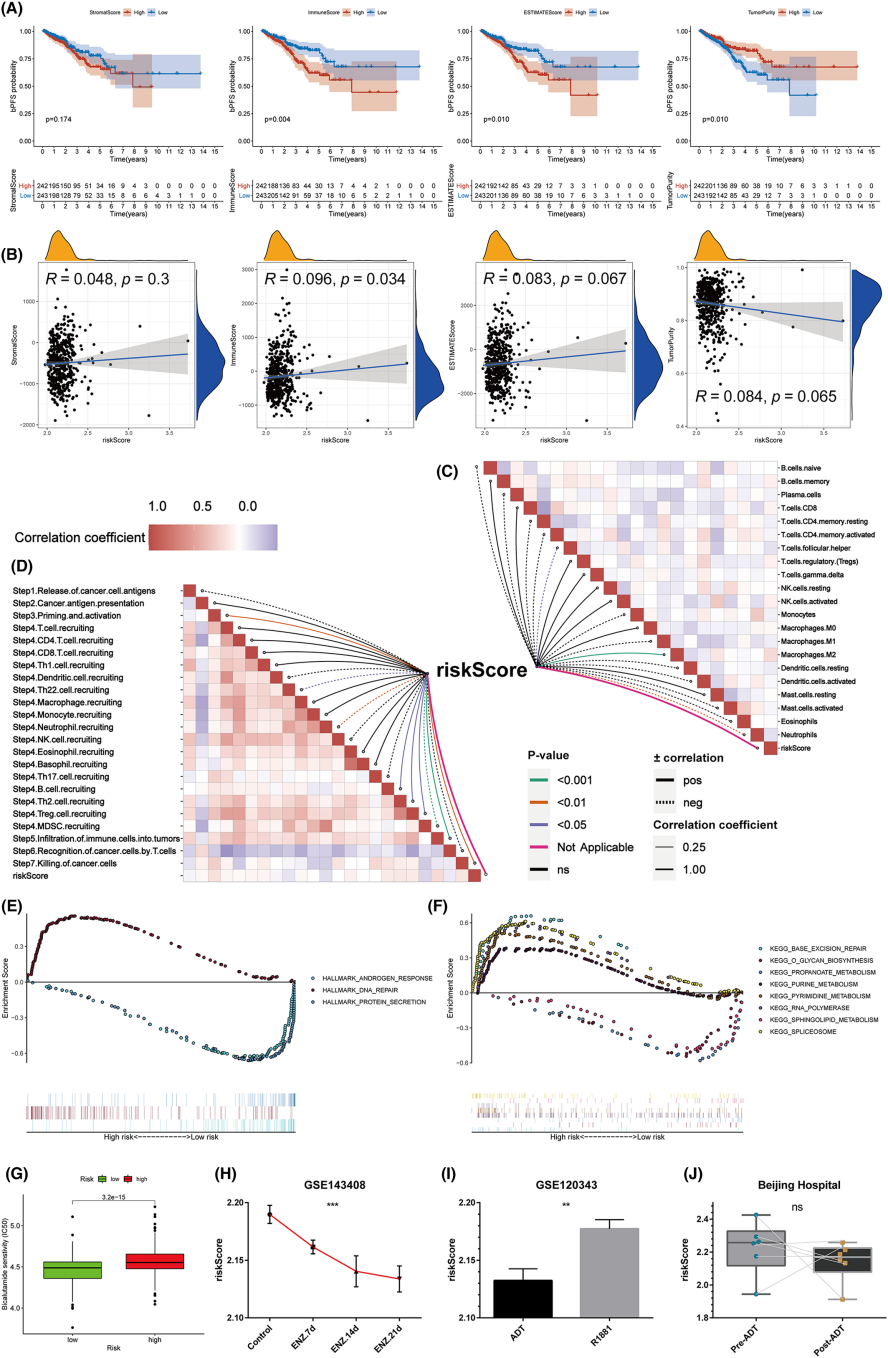
Immune characteristics and GSEA. (A) Kaplan–Meier survival analysis of the StromalScore, ImmuneScore, ESTIMATEScore, and TumorPurity. (B) The correlation between signature risk score and the ESTIMATE results. (C) The correlation between signature risk score and immune cell infiltration. (D) The correlation between signature risk score and the seven‐step cancer immune cycle. (E) GSEA of the Hallmark pathway between high‐ and low‐ risk groups. (F) GSEA of the KEGG pathway between high‐ and low‐ risk groups. (G) Using the “pRRophetic” package to predict the sensitivity of patients to bicalutamide. (H) Signature risk score decreased as the processing time of enzalutamide increased in LNCaP cell lines. (I) Signature risk score of ADT‐treat group was significantly lower than that of androgen stimulation group in LNCaP cell lines. (J) Signature risk score of post‐ADT radical prostatectomy samples was decreased compared with pre‐ADT biopsy samples.

An ever increasing number of studies have shown that tumor metabolic reinventing is related to immune infiltration and immune response.^18^, ^19^ The ESTIMATE algorithm was used to obtain the StromalScore, ImmuneScore, ESTIMATEScore, and TumorPurity of each patient. Survival analysis showed that different ImmuneScore, ESTIMATEScore, and TumorPurity had different bPFS probability (*p* < 0.05) (Figure 8A). Then we explore the correlation between our signature risk score and the ESTIMATE results and found that only the ImmuneScore and the signature risk score had a statistically significant correlation (*p* < 0.05) (Figure 8B). Therefore, we have more reason to believe that this signature is related to immune function and immune cell infiltration. The results showed that the risk score was negatively correlated with T cells follicular helper and neutrophils, but positively correlated with macrophages M2 (*p* < 0.05) (Figure 8C) (Table S7). When exploring the relationship of the risk score with the seven‐step cancer immune cycle, we found that the risk score was positively correlated with priming and activation (Step 3), B/Th2/Treg recruiting (Step 4), infiltration of immune cells into tumors (Step 5) and killing of cancer cells (Step 7), but negatively correlated with Th22/Neutrophil/MDSC cell recruiting (Step 4) (*p* < 0.05) (Figure 8D) (Table S8).

### 3.8 GSEA between different risk groups

To clarify the molecular mechanism associated with the signature, GSEA software was utilized to track down the differences between the low‐and high‐ risk groups in the Hallmark and KEGG pathway. The high‐risk group was associated with DNA repair and the low‐risk group was associated with androgen response and protein secretion in the Hallmark analysis (Figure 8E). In the KEGG analysis, the top KEGG terms enriched in the high‐risk group were spliceosome, base excision repair, pyrimidine metabolism, RNA polymerase, and purine metabolism. For the low‐risk group, the top KEGG terms enriched were O glycan biosynthesis, propanoate metabolism, and sphingolipid metabolism (Figure 8F) (Table S9). Here, we observed that in addition to metabolic pathways and biological processes, the low‐risk group enriched androgen response, which aroused our great interest. Androgen deprivation therapy (ADT) is the foundation of the treatment of advanced PCa. This may suggest that the signature may predict the response of PCa patients to ADT. To test our conjecture, we first used “pRRophetic” package to predict the sensitivity of patients to bicalutamide. The estimated IC50 value indicated that the low‐risk group had a superior response to the bicalutamide (*p* < 0.001) (Figure 8G). Next, we used two cytological data sets (GSE143408 and GSE120343) and our local patient data set (Beijing Hospital) to verify the conjecture again. LNCaP cells were treated with enzalutamide in GSE143408 data set, and then collected at 0, 7, 14, and 21 days for microarray analysis.^13^ We found that our signature risk score decreased as the processing time of enzalutamide increased (Figure 8H). LNCaP cells were also used in GSE data set. The experimental group was treated with ADT, while the control group was treated with synthetic androgen R1881 and then used for microarray analysis.^14^ The results showed that the risk score of ADT group was significantly lower than that of androgen stimulation group (Figure 8I). Finally, six PCa patients from our local hospital received transcriptome sequencing on pre‐ADT biopsy samples and post‐ADT radical prostatectomy samples. Sequencing data has been uploaded to the GEO platform (GSE150368). Overall, patients' risk scores decreased significantly after ADT treatment (Figure 8J). However, after careful analysis of the paired comparison of each patient, it was found that the risk score of three patients increased instead after receiving ADT treatment. We explored the reasons and traced the clinical information, and found that the ADT treatment effect of these three patients was not ideal, and the nadir PSA value after ADT was all higher than 0.2 ng/ml. In contrast, the other three patients with decreased risk score had very good ADT treatment effect, and all nadir PSA levels were lower than 0.1 ng/ml (Table S10). Of course, due to the small sample size, it needs to be confirmed in the subsequent real‐world studies.

## 4 DISCUSSION

PCa cases in the world are increasing, the incidence in Asia, Nordic and Western Europe has risen sharply.^20^ Most patients with PCa can benefit from radical treatment and obtain relatively good prognosis. However, patients with advanced PCa often have poor prognosis due to BCR or distant metastasis.^21^ BCR is defined as PSA rising to more than 0.2 ng/ml again, which is confirmed by two consecutive rising values. It is a decisive risk factor for distant metastasis, cancer specificity, and overall mortality.^22^, ^23^ Therefore, looking for new biomarkers for PCa BCR with higher prediction accuracy has important clinical application value.

As the result and cause of cancer formation, metabolism in tumor cells plays an important role in tumorigenesis.^24^ Unlike normal cells, cancer cells reengineer their cellular metabolism to promote growth and survival, and many different types of cancer show similar metabolic changes.^25^ Most solid tumors experience Warburg effect, whereby malignant cells transfer their main ATP production pathway from oxidative phosphorylation to aerobic glycolysis.^26^ While glycolysis is not as efficient as aerobic respiration in terms of energy supply, it produces ATP 100 times faster than mitochondrial oxidation, which helps maintain the high energy levels needed for tumor development. This feature explains the success of 18‐fluoro‐2‐deoxyglucose positron emission tomography (FDG‐PET) in imaging multiple histological types of tumors. However, PCa cell metabolism is unique, even completely opposite to the metabolic phenotype of most other tumors. Benign prostatic epithelial cells inhibit citrate oxidation through high accumulation of zinc and promote citrate synthesis and secretion as a major component of prostatic fluid to nourish and protect sperm. In contrast, PCa cells adopt zinc deficiency, reduced citric acid secretion and transition to the citric acid oxidation phenotype, enabling PCa cells to utilize the tricarboxylic acid cycle and subsequent oxidative phosphorylation.^27^, ^28^ Therefore, the Warburg effect is not significant in PCa because these cells do not have increased glucose uptake. This also explains why FDG‐PET is not routinely used in clinical imaging of PCa. Only in the late stage with a large number of mutation events, PCa will show Warburg effect and high glucose intake. In addition, PCa is closely related to androgen and androgen receptor (AR), which is also different from other tumors. Multiple omics studies have clearly demonstrated that AR is the master coordinator of cellular energy metabolism. AR regulates many metabolic pathways in PCa, including glycolysis, mitochondrial respiration, fatty acid oxidation, steroid, and other lipid synthesis, nutritional reorganization between glycolysis and mitochondrial respiration, and amino acid uptake and consumption, which clearly defines AR as an important driver of PCa cell metabolism.^29^ In view of the above unique metabolic characteristics of PCa, it is feasible and expected to predict the prognosis of PCa from metabolic characteristics.

This study was based on TCGA‐PCa data sets, which determined a robust risk score model of four genes of POLR2F, CPT1B, MTHFD1L, and ASNS. And the signature is confirmed by both the validation and the test set. The results show that high‐risk group patients are closely related to poor prognosis. All of these genes have been proven to be related to tumors in previous studies. POLR2F encodes the sixth largest subunit of RNA polymerase II, the polymerase responsible for synthesizing messenger RNA in eukaryotes. Anna G Antonacopoulou et al.^30^ believe that it may be a prognostic marker molecule in colorectal cancer, but there are few studies focusing on this gene in tumors at present. Abudurexiti M et al.^31^ found targeting CPT1b as a potential therapeutic strategy in CRPC. Studies have found the downregulation of CPT1B can cause fatty acid β‐oxidation impairment and play an important role in the high‐grade progression of bladder cancer.^32^ MTHFD1L has metabolic advantage in liver cancer and can be used as a potential tumor marker of liver cancer.^33^, ^34^ MTHFD1L is also involved in the progression of esophageal cancer, bladder cancer, colorectal cancer, and tongue cancer.^35^, ^36^, ^37^, ^38^, ^39^ The synthesized MTHFD1L shRNA nanoparticles can be used to treat oral cancer.^40^ ASNS gene encodes an enzyme (Asparagine synthetase) that is responsible for catalyzing the conversion of aspartic acid to asparagine. The downregulation of ASNS can inhibit the proliferation of breast cancer and gastric cancer cells,^41^, ^42^ and its upregulation increases the sensitivity of nasopharyngeal carcinoma cells to cisplatin.^43^ Targeted ASNS inhibitors may be a new strategy for targeting CRPC.^44^ The expression of ASNS is also an independent predictor of postoperative survival of hepatocellular carcinoma.^45^ It is worth mentioning that the coefficient of POLR2F gene is significantly higher than that of the other three genes involved in the construction of features (Figure 4D). A greater weight usually indicates that this gene is more important in prognosis prediction. In the TCGA dataset, POLR2F was co‐expressed with 316 metabolic genes, which was 343 in the MSKCC dataset and 60 in the GSE70769 dataset (Table S11). From the perspective of biological function, the increased expression of POLR2F may reflect the high transcriptional activity in tumor cells. Therefore, high transcriptional activity may be the cause of overexpression of other important metabolic genes involved in tumor progression. Of course, this causal relationship needs further verification in the future.

Several studies have shown that metabolic reprogramming can have a significant impact on the tumor microenvironment.^46^, ^47^ We found that patients with high immune score had worse bPFS, which was statistically correlated with our signature risk score. Immune cells are an important part of tumor microenvironment,^48^ and anti‐tumor immune function is an important barrier against tumor.^49^ Our results showed that the risk score was associated with only a few immune cell infiltrates and only a few relevant anti‐tumor immune functions. It may be related to the fact that PCa is usually an inert and “cold” tumor with relatively low mutation burden and minimal T cell and immune invasion.^50^

The biological processes of the high‐ and low‐risk score groups were analyzed by GSEA. Results have shown that the most up‐regulated part of KEGG gene sets were mainly related to the pathways related to cell metabolism in both high‐risk and low‐risk populations. When exploring Hallmark gene set, we found that low‐risk population was significantly enriched in “Androgen response.” The results of subsequent cytological experiments and a population cohort receiving ADT with pre‐ADT/post‐ADT transcriptome information fully prove a conjecture that the metabolic signature can effectively predict the response of patients to ADT. Indeed, ADT not only affects PCa, but also affects a variety of metabolism, including hematopoiesis, bone, lipid, protein, nucleic acid, and glucose metabolism,^51^, ^52^ and even leads to metabolic syndrome.^53^ Therefore, ADT may reduce the risk score of patients after ADT by affecting the expression of metabolic genes in the signature.

This study synthesized and analyzed high‐throughput sequencing data from multiple databases, constructed signature based on MRGS, and gradually established personalized nomograph to predict BCR, which was verified by independent cohort. But there are still some limitations in our study. This study is a retrospective study and further prospective results are needed to support each other. For this prognostic signature to predict patients' response to ADT treatment, it has only been preliminarily verified by a small sample size, and further verification of a larger sample size is needed in the future. The association between metabolic genes in the signature and tumor development is unknown, and the detailed mechanism needs to be explored with the help of in vivo and in vitro validation experiments, especially the effects of POLR2F on tumor transcriptional activity and the regulation of other metabolic gene expression.

## 5 CONCLUSION

In conclusion, we established a metabolism‐related risk signature based on four genes. We also explored its relationship with immune cell infiltration and anti‐tumor immunity, which can be used as a tool to predict the clinical prognosis and guide the ADT treatment of PCa patients.

## AUTHOR CONTRIBUTIONS

Zhengtong Lv, Xiaoxiao Guo, and Haoran Xia: Data curation, Formal analysis, Software, Validation, and Visualization. Jianlong Wang, Xuan Wang, and Ming Liu: Funding acquisition, Investigation, Project administration. Jianye Wang: Project administration, Supervision, and Writing – review & editing. Zhengtong Lv: Conceptualization, Investigation, and Writing – original draft.

## FUNDING INFORMATION

This work was funded by BJ‐2020‐171 and BJ‐2018‐090 from Beijing Hospital Clinical Research 121 Project and grants 3332019122 and 3332020069 from the Fundamental Research Funds for the Central Universities.

## CONFLICT OF INTEREST

The authors declare that they have no competing interests.

## ETHICS STATEMENT

Study involving human participant was reviewed and approved by the Ethics Committee of the Beijing Hospital (2018BJYYEC‐085‐03). The patient provided his written informed consent to participate in this study.

## Supporting information

Table S1
Table S2
Table S3
Table S4
Table S5
Table S6
Table S7
Table S8
Table S9
Table S10
Table S11

## Data Availability

The datasets generated and/or analyzed during the current study are available in the TCGA repository (https://portal.gdc.cancer.gov/), the MSKCC database (http://cbio.mskcc.org/cancergenomics/prostate/), and GSE70769, GSE143408 and GSE120343 from GEO repository (https://www.ncbi.nlm.nih.gov/geo/).
